# Development of an *in vivo* technique for dose verification at the prone breast board / skin interface

**DOI:** 10.1002/acm2.13229

**Published:** 2021-03-24

**Authors:** Brian Loughery, Dennis Chan, Jay Burmeister, Michael Dominello

**Affiliations:** ^1^ Department of Oncology Wayne State University School of Medicine Detroit MI USA; ^2^ Department of Radiation Oncology Karmanos Cancer Institute Detroit MI USA; ^3^ Department of Radiation Oncology Stritch School of Medicine Cardinal Bernardin Cancer Center Loyola University Chicago Maywood IL USA

**Keywords:** breast board, *in vivo* dosimetry, prone breast

## Abstract

Due to the limited height of commercial prone breast boards, large or pendulous breasts may contact the base layer of the board during simulation and throughout the course of treatment. Our clinic has historically identified and marked this region of contact to ensure reproducible setup. However, this situation may result in unwanted hotspots where the breast rests atop the board due to electron scatter. In this study, we performed *in‐vivo* dosimetric measurements to evaluate the surface dose in regions of contact with the immobilization device. The average dose and hotspot were identified and evaluated to determine whether plan modifications were necessary to avoid excess skin toxicity at the skin/breast board interface. The film method results were validated against a commissioned *in vivo* OSLD dosimetry system. Radiochromic film measurements agreed with OSLD readings (n = 18) overall within 1%, σ = 6.4%, with one deviation of >10%. Pertinent information for the physician includes the average, maximum, and minimum doses received at the film interface. Future readings will not require OSLD verification. Physicians now have access to additional spatial data to correlate skin toxicity with doses delivered at the skin/breast board interface. This new technique is now an established procedure at our clinic, and can inform future efforts to model enhanced methods to calculate the dosimetric effects from the prone breast board in the treatment planning system.

## 1 INTRODUCTION

**Fig. 1:**
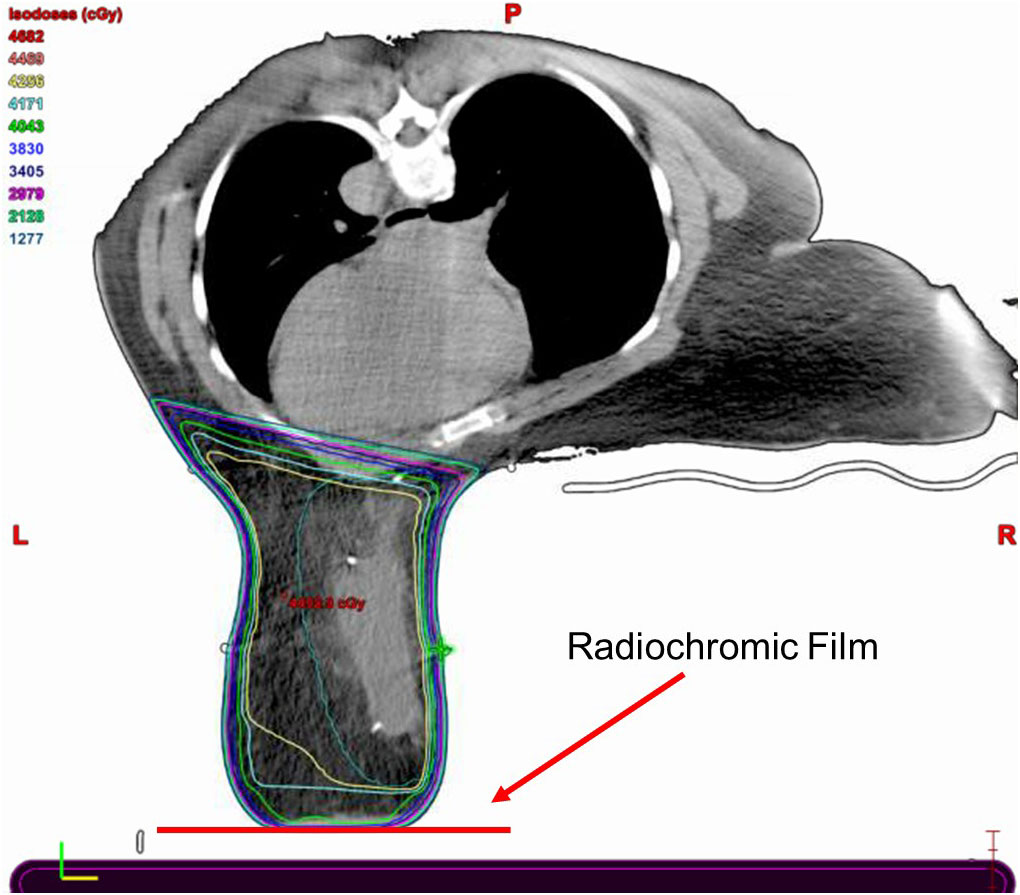
“Prone Breast CT Slice.” Slice of prone breast treatment plan, demonstrating an intended reduction in absorbed dose near the breast board/skin interface.

Radiation therapy is an essential component of postlumpectomy treatment for breast cancer patients and can be delivered with patients either in the supine or prone position. Treatment in the prone position takes advantage of gravity and allows the breast, and hence the target, to fall away from the chest wall, ipsilateral lung, and heart. Prone breast tangent fields generally avoid primary beam interaction with the prone breast board; however, for patients where the breast contacts the base layer of the prone breast board, the primary beam must pass through the base layer to treat the entire breast (Fig. 1). This results in largely unaccounted for electron scatter that usually increases the surface dose and is difficult to accurately model in the treatment planning system (TPS). An *in vivo* dose measurement at the prone breast board/skin interface is warranted to accurately assess the dose to the surface of the breast.

**Fig. 2:**
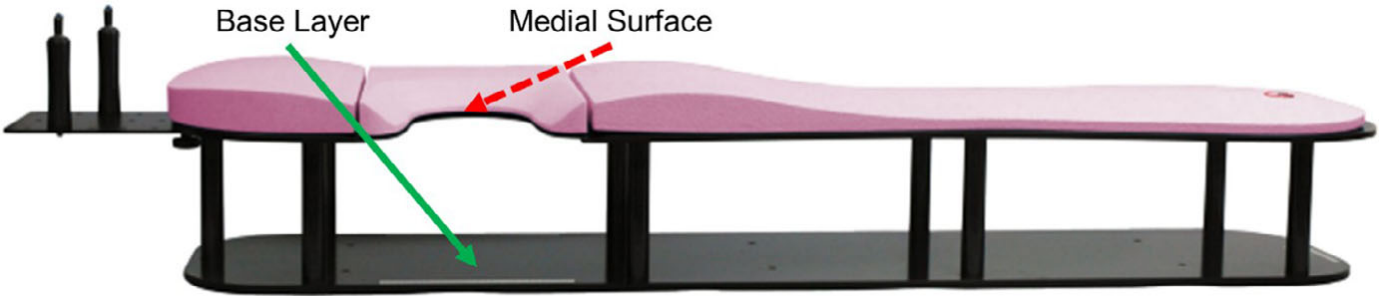
“Prone Breast Board.” Prone breast board used in this study. This study measures the enhanced skin dose from the anterior contact surface of the breast with the base layer (solid green arrow) as opposed to the medial contact surface (dashed red arrow). Breast board image from Qfix.^10^

The American Association of Physicists in Medicine (AAPM) Task Group (TG)‐176 recommends fully modeling couchtop structures and immobilization devices in the TPS or to avoid irradiating through couchtop immobilization devices.^1^ Several subsequent studies have investigated dosimetric impacts of the prone breast board in general.^2^, ^3^, ^4^ While previous studies have investigated loss of skin sparing due to contact with the prone breast board, they are not sufficient to evaluate and address the clinical problem described here. A recent study rigorously measured couch rail and prone breast board transmission factors, finding that a clinical prone breast case generally receives less dose than the TPS predicts in the absence of proper modeling.^3^, ^4^ Film studies were performed primarily through the sides of the prone breast board and directly through the couch structure. Surface dose measurements were underpredicted by the TPS on the order of 25–35%.^3^, ^4^ These studies were performed in a phantom and used ion chamber measurements in a solid water block, but were not performed *in vivo* and did not measure surface dose against the anterior contact surface of the breast, focusing instead on the medial edge of the breast at the chest wall (Fig. 2). Further work has been limited to radiochromic film measurements at the medial edge of the prone breast board in phantom^5^ and a case study using an anthropomorphic phantom to measure contact with the inferior and medial edge contact points near the inframammary fold.^6^

The purpose of this technical report is to describe our method for measuring dose across the contact plane between the breast and the base layer of the prone breast board. The report also describes our verification of this method using an established *in vivo* dosimetry system. The procedure detailed below is now clinically implemented for all prone breast patients whose treated breast contacts the base layer of the prone breast board. The information from this evaluation serves either to assure the physician that the treatment plan sufficiently minimizes risk of skin toxicity, or to guide the physician to request a new treatment plan designed explicitly to reduce the surface dose while maintaining all other planning goals.

## 2 MATERIALS AND METHODS

**Fig. 3:**
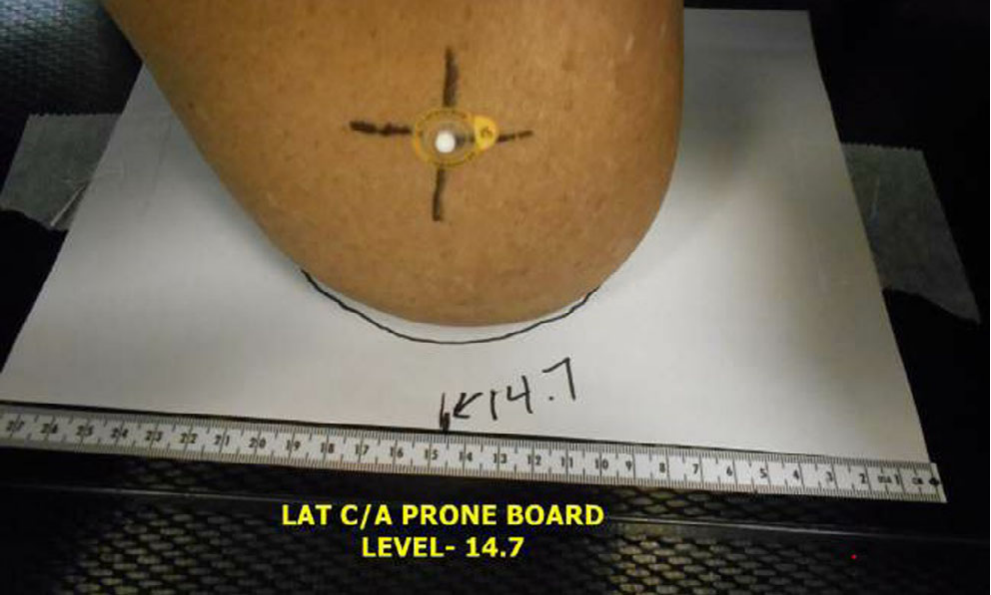
“Setup Trace Paper” Patient setup trace paper in place during typical setup. his paper is replaced with radiochromic film for *in vivo* measurement.

During CT simulation for prone breast treatments in our clinic, we identify areas where the breast is in contact with the base layer of an Access ClearVue prone breast board (Qfix, Avondale, PA) by indexing a piece of white printer paper to the base layer under the breast (Fig. 3). Once the breast is positioned in a reproducible location, the contact plane is traced with a marker and the indexed position is recorded. The setup trace paper is in place for all fractions to guide radiation therapists toward a reproducible setup. Image guidance is performed for the first fraction, then every five subsequent fractions.

For this study, we replaced the setup trace paper with Gafchromic EBT3 (Ashland, Bridgewater, NJ) radiochromic film. Prior to the patient’s second fraction, the physicist copies the entire setup trace onto a piece of EBT3, which is then used by the radiation therapists in place of the setup paper. We chose fraction #2 for dosimetric evaluation to eliminate dose contamination from pretreatment imaging. Two strips are cut from the short edge of the film prior to irradiation for use with the one‐scan protocol.^7^ One strip is irradiated to 120% of the expected dose to the film surface as predicted in the TPS, and the other is left unirradiated. Both film strips and the treatment film are scanned into an Epson 10000XL scanner set to 0.0 focus, 48‐bit depth, and 72DPI resolution. Analysis is performed in FilmQA Pro (Ashland, Bridgewater, NJ) calibrated with tri‐color optimization. Care is taken throughout the process to ensure that films and strips are scanned in a consistent alignment. All films are scanned the day after treatment to ensure that sufficient time was given for the film to polymerize.

**Fig. 4:**
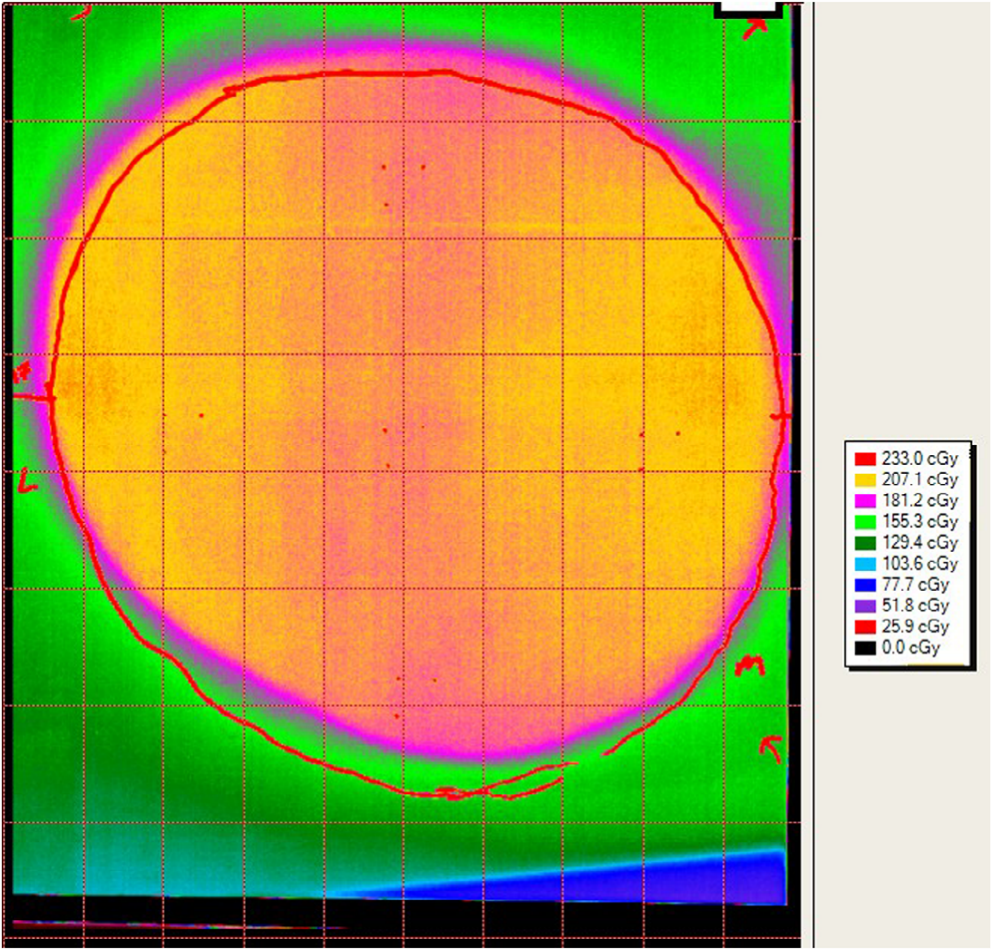
“Example Film Readout” Calibrated film readout, showing patient trace (red outline) and dose distribution.

A report is created for the physician with a custom standardized isodose map that is designed to be as similar as possible to the default isodose lines in the TPS (Fig. 4). The report highlights the average dose and maximum point dose, given the scanned resolution (~0.3 mm × 0.3 mm). These doses are displayed as a percentage of the prescribed dose to the breast.

**Fig. 5:**
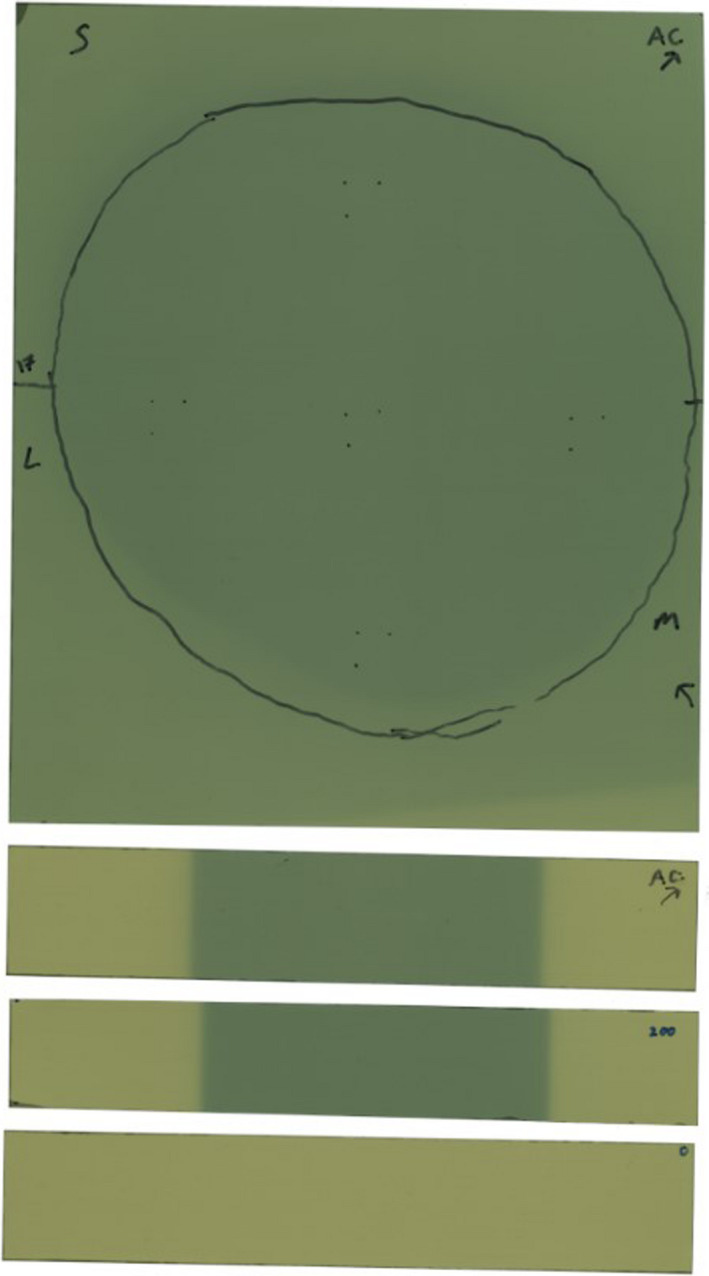
“Example Film Scan” Scanned film prior to readout, showing patient trace (black outline) and calibration strips. Markings for five OSLDs can be seen at center and cardinal directions.

This methodology was validated against our established *in vivo* dosimetry program, which uses nanoDot (Landauer Inc, Glenwood, IL) Optically Stimulated Luminescent Dosimeters (OSLDs). For this validation we taped one to four OSLDs, based on breast contact plane size, to the radiochromic film. Small opaque marks were made on the film at the three nondetecting corners of each OSLD (Fig. 5). Point doses measured in the detecting area were compared to the film doses. Our OSLD system was commissioned in accordance with the high‐accuracy method of AAPM TG‐191,^8^ except that our angular correction factors (k_θ_) were measured in phantom to be 1.03 and applied manually.^9^ OSLD standards were given the same dose as the irradiated film strip (above). To provide sufficient validation of the film dosimetry technique, the first seven instances of this protocol included OSLD verification measurements. It should be mentioned that any clinical decisions made as a result of this protocol were drawn from the established OSLD readings and not from the novel film measurements listed in Table 1.

**Table 1:** Data from all patients enrolled in this study, including measured hotspots.

Reading	Average film/OSLD	OSLDs used	Breast CTV (cc)	Maximum % of prescribed dose
1^b^	0.922	2	1629	0.748
2^a^, ^b^	0.951	4	2526	1.071
3	1.014	4	2526	1.131
4^b^	1.127	2	1557	1.195
5^b^	1.010	2	3484	1.221
6	0.982	1	3350	1.224
7	1.007	3	2458	1.267
Average:	1.002 ± 0.064			1.123 ± 0.177

^a^ Replan of reading 3.

^b^ Deliberately planned to <95% Rx at breast board/skin interface.

## 3 RESULTS AND DISCUSSION

We performed this evaluation eight times on seven patients. The first patient was a proof‐of‐concept without OSLDs that does not resemble the other measurements, so it is excluded from this study. One patient was replanned and remeasured, leading to a total of seven readings from six patients used for the analysis presented here. These seven readings resulted in a total of 18 OSLD data points and seven sheets of film.

Table 1 summarizes the validation of the film method, noting the replanned measurement with ^a^. A direct comparison of film readings to OSLD readings is provided — a value greater than 1 demonstrates a film measurement that is greater than what was measured on an OSLD at the same location. The mean film result is within 0.2% of the mean OSLD result, with a standard deviation of 6.4%. All film‐to‐OSLD comparisons were within the stated error of unscreened nanoDot OSLDs (10%) except for one measurement in patient 4, which skewed the average for that patient above 10%. Patient 4 is the only subject with film measurements that exceed 8% deviation from the corresponding OSLDs.

Hotspots found in the film are listed as a percentage of the prescription dose. Dose was prescribed to the entire breast volume and varied from 180 to 266 cGy/fx. Film hotspots were found to be higher than the prescription dose in six of seven cases. In the patients marked with ^b^, a field‐in‐field technique was used to shield the breast board/skin interface to maximum of 95% of the prescription dose. For example, reading 2 directly applies field‐in‐field shielding to the interface to the plan from reading 3, leading to a 6%‐of‐prescription improvement in the hotspot. Despite this effort, the average hotspot observed on the films was 112% of prescription. This is a troubling result without an obvious cause — it is possible that our method of prone breast treatment planning is not well modeled in the TPS due to the heterogeneity interface between the breast board and the breast. Another working theory was that it was somehow correlated with CTV volume, but we found no correlation between hotspot reading and CTV volume (R^2^ = 0.3).

The major limitation of this study is that we only had seven eligible patients due to our use of the ClearVue, which boasts a large gap between the chest wall and the base layer of the board. Contact with the base layer an uncommon occurrence at our clinic, as is incidental dose to the heart. More attention should be paid to incidental heart dose if repeating this technique with a smaller prone breast board. We also only repeated our technique in one patient. Our methodology is intended to be used early in a treatment course so that the plan can be adapted to compensate for a detected overdose at the interface.

## 4 CONCLUSION

We have developed and validated a simple radiochromic film technique for measurement of surface dose for prone breast treatments in which the breast contacts the prone breast board. Film measurements guarantee that the hotspot at the contact plane will be measured, and when compared to OSLDs are free of directional dependence and the need to track several individual dosimeters. Preliminary results have led to replanning, additional field‐in‐field shielding of the interface for all future cases, and a change in clinical practice that includes dose verification for each such patient undergoing prone breast radiotherapy. Future work will include analysis of our results with respect to the TPS to observe and quantify patterns in the ability of the TPS to appropriately model scatter into the breast from the base layer of the prone breast board. This can be measured against a prone breast board without a base layer, such as the Qfix Prone G2. This novel application of radiochromic film for adaptive replanning is now established practice at our clinic and has anecdotally resulted in decreased acute skin toxicity for this patient population.

## AUTHOR CONTRIBUTION STATEMENT

Dr. Loughery: Lead author of manuscript, physics data collection and analysis, corresponding author. Dr. Chan: Coauthor of manuscript, clinical data collection and analysis. Dr. Burmeister: Study design, coauthor of manuscript, data analysis, and physics expertise. Dr. Dominello: Lead study design, data analysis, senior author of manuscript, and clinical expertise.

## References

[1] Olch AJ , Gerig L , Li H , et al. Dosimetric effects caused by couch tops and immobilization devices: report of AAPM Task Group 176. Med Phys. 2014;41:061501. 24877795 10.1118/1.4876299

[2] Yoo S , Horton JK Sr , Yin FF , et al. Dosimetric effect of the breast board and couch top for whole‐breast radiation therapy in the prone position. Int J Radiat Oncol Biol Phys. 2016;96:E46–E47.

[3] Lau AJ . Dosimetric Impact of Immobilization Board and Couch Structures in Prone Breast Radiation, University at Buffalo; 2017.

[4] Wang I . Presented at the Radiation Oncology Conference for Therapists and Dosimetrists, Niagara Falls, NY; 2016. (unpublished).

[5] Guerra M , Jozsef G . SU‐F‐T‐93: Breast Surface Dose Enhancement Using a Clinical Prone Breast Board; 2016. 10.1118/1.4956229

[6] Gonzalez V , Gloss J . (P03) Impact of breast board position on skin dose in patients receiving prone breast radiotherapy. Int J Radiat Oncol Biol Phys. 2018;101:E21–E22.

[7] Lewis D , Micke A , Yu X , et al. An efficient protocol for radiochromic film dosimetry combining calibration and measurement in a single scan. Med Phys. 2012;39:6339–6350. 23039670 10.1118/1.4754797 PMC9381144

[8] Kry SF , Alvarez P , Cygler JE , et al. AAPM TG 191: clinical use of luminescent dosimeters: TLDs and OSLDs. Med Phys. 2020;47:e19–e51. 31574174 10.1002/mp.13839

[9] Kerns JR , Kry SF , Sahoo N , et al. Angular dependence of the nanoDot OSL dosimeter. Med Phys. 2011;38:3955–3962. 21858992 10.1118/1.3596533 PMC3133805

[10] Qfix . Access ClearVue Prone Breast Device; 2017. Available at https://qfix.com/catalog/radiotherapy‐breast‐torso‐solutions‐prone‐positioning/access‐clearvue‐prone‐breast‐device

